# Population pharmacokinetic and exposure–response analysis for trastuzumab administered using a subcutaneous “manual syringe” injection or intravenously in women with HER2-positive early breast cancer

**DOI:** 10.1007/s00280-015-2922-5

**Published:** 2015-12-08

**Authors:** Angelica L. Quartino, Carina Hillenbach, Jing Li, Hanbin Li, Russell D. Wada, Jennifer Visich, Chunze Li, Dominik Heinzmann, Jin Y. Jin, Bert L. Lum

**Affiliations:** Genentech, Inc., California, CA USA; Roche, Basel, Switzerland; Quantitative Solutions, Inc., Menlo Park, CA USA

**Keywords:** Early breast cancer, Fixed dose, HER2, NONMEM, Population pharmacokinetics modeling, Trastuzumab

## Abstract

**Purpose:**

To characterize the population pharmacokinetics (PKs) of subcutaneous (SC) and intravenous (IV) trastuzumab in early breast cancer (EBC), assess the impact of covariates on trastuzumab PK, and evaluate fixed (nonweight-based) dosing for the SC regimen administrated via handheld syringe.

**Methods:**

Serum trastuzumab concentrations from 595 patients with HER2-positive EBC in the HannaH study (fixed 600 mg SC trastuzumab or weight-based IV trastuzumab) were analyzed using nonlinear mixed-effects modeling. Multiple logistic regression was used to assess the exposure–response relationships between PK, efficacy [pathologic complete response (pCR)], and safety [grade ≥3 adverse events (AEs)].

**Results:**

Trastuzumab PK was described by a two-compartment model with parallel linear and nonlinear elimination and first-order SC absorption, with a bioavailability of 77 %. Estimated total clearance (CL) values were 0.18–0.22 L/day for steady-state trough/peak concentrations of 75–148 µg/mL; the estimate for central volume of distribution was 2.9 L. Body weight and alanine transaminase, while showing significant effects on PK, only explained 8 % of the variability in CL. Exposure–response analyses showed no relationship between PK, pCR, and grade ≥3 AEs for either regimen.

**Conclusion:**

A fixed 600 mg SC dose of trastuzumab provides the desired exposure, with steady-state trough concentrations (35–123 μg/mL for the 5th–95th percentiles) above the historical target concentration of 20 μg/mL for efficacy. Fixed dosing is further supported by lack of an exposure–response relationship between PK, pCR, and grade ≥3 AEs. No dose adjustment per patient factors is required within the ranges studied.

**Electronic supplementary material:**

The online version of this article (doi:10.1007/s00280-015-2922-5) contains supplementary material, which is available to authorized users.

## Introduction

Trastuzumab is a humanized immunoglobulin G1 (IgG1) monoclonal antibody against the extracellular domain of human epidermal growth factor receptor 2 (HER2) [1, 2]. Trastuzumab is currently licensed in the USA/EU for treatment of patients with HER2-positive early breast cancer (EBC) (in combination with neoadjuvant and/or adjuvant chemotherapy), HER2-positive metastatic breast cancer (MBC) (as monotherapy or in combination with pertuzumab plus docetaxel, or with lapatinib, paclitaxel, docetaxel, or an aromatase inhibitor), and HER2-positive metastatic gastric cancer (in combination with capecitabine or 5-fluorouracil and cisplatin) [3, 4]. The approved intravenous (IV) regimens for breast cancer include an 8 mg/kg loading dose followed by 6 mg/kg maintenance doses administered every 3 weeks (q3w) or a 4 mg/kg loading dose followed by a 2 mg/kg weekly (qw) maintenance dose [3, 4]. IV administration can be time-consuming and may result in patient discomfort, whereas a subcutaneous (SC) fixed-dose administration can be delivered in <5 min without IV access, thereby increasing the overall convenience of trastuzumab administration [5]. A 600 mg fixed SC dose of trastuzumab, administered by manual injection/handheld syringe q3w, has been approved in the EU and in a number of non-EU states (Argentina, Chile, Costa Rica, Macedonia, Norway, Panama, Peru, South Korea, and Uruguay) [4, 6]. This administration contains recombinant human hyaluronidase (rHuPH20), which temporarily degrades hyaluronan at the injection site to facilitate injection of the 5 mL dosing volume [7].

The pharmacokinetics (PKs) of IV trastuzumab were previously characterized in patients with HER2-positive MBC using a population PK (PopPK) model [8]. Here, a two-compartment model with linear clearance (CL) was sufficient to describe the PK of trastuzumab [8, 9]. Body weight was an important covariate, with increased CL and central volume of distribution (*V*_c_) in heavier patients [8, 9]. To identify the SC dose for the HannaH study, a PopPK model for SC trastuzumab was developed based on a phase I study (NCT00800436), in which abundant PK data were obtained from 24 healthy volunteers and 42 patients with HER2-positive EBC receiving single, weight-adjusted doses of SC and/or IV trastuzumab [5]. A PopPK model consisting of a two-compartment model with parallel linear and nonlinear (Michaelis–Menten) elimination from the central compartment was then used to verify that a fixed-dose SC regimen of 600 mg would achieve a trough concentration (*C*_min_) noninferior to the IV regimen [10]. The nonlinear CL has been reported for other monoclonal antibodies and is thought to reflect target-mediated drug disposition [11]. In addition, the concentrations for the 600 mg SC dose are expected to consistently exceed the historical efficacy *C*_min_ target of 20 μg/mL identified in preclinical studies [12].

HannaH (NCT00950300) was a phase III, randomized, international, open-label trial in the neoadjuvant–adjuvant setting, conducted to establish noninferiority of fixed-dose SC trastuzumab with the approved weight-based IV q3w regimen [13]. Patients with HER2-positive, operable, locally advanced, or inflammatory breast cancer were randomized 1:1 to receive eight cycles of neoadjuvant chemotherapy and concurrent trastuzumab q3w, administered either IV (8 mg/kg loading dose, 6 mg/kg maintenance dose) or SC (600 mg fixed dose). Following surgery, patients continued trastuzumab q3w to complete 1 year of adjuvant therapy. *C*_min_ and pathologic complete response (pCR) were coprimary endpoints. The geometric mean ratio of presurgery (precycle 8) *C*_min_ in the SC and IV arms was 1.33, with two-sided 90 % confidence interval (CI) of 1.24–1.44, the lower limit of which was higher than the prespecified noninferiority margin of 0.80. The pCR rates were 40.7 and 45.4 % in the IV and SC arms, respectively, a difference of 4.7 % (95 % CI −4.0, 13.4); the lower limit of the two-sided 95 % CI was higher than the prespecified noninferiority margin of −12.5 %. Thus, noninferiority of SC versus IV trastuzumab was demonstrated for both endpoints.

We present results from the PopPK analysis of HannaH. The key objectives of this analysis were (1) to characterize the PopPK of trastuzumab in the SC “manual syringe” injection and IV administrations, (2) to assess the effect of patient characteristics on trastuzumab PK, (3) to explore the exposure–response relationship between PK, pCR, and grade ≥3 adverse events (AEs) for the SC regimen, and (4) to evaluate further the appropriateness of a fixed 600 mg SC dose.

## Patients and methods

### Patients

HannaH enrolled 596 patients with HER2-positive EBC, 595 of whom received at least one dose of trastuzumab. Patients were randomized to receive either SC (fixed 600 mg SC dose; *n* = 297) or IV (8 mg/kg IV loading dose, 6 mg/kg IV maintenance dose; *n* = 298) trastuzumab (Herceptin^®^, F. Hoffmann-La Roche, Basel, Switzerland) q3w, for a total of 18 cycles over 1 year of therapy (Online Resource 1). Briefly, all patients received concomitant chemotherapy during the first eight presurgery cycles (neoadjuvant phase) and trastuzumab monotherapy for the remaining ten post-surgery cycles (adjuvant phase). SC trastuzumab was injected with a manual handheld syringe over 5 min; IV trastuzumab was administered as an initial 90-min infusion with subsequent 30-min infusions. Safety analyses were performed after approximately 20 months of follow-up [14]. The study was conducted in accordance with the Declaration of Helsinki, and the protocol and all amendments thereof were approved by an independent ethics committee. Participants provided written informed consent.

### PK data included in the analysis

Blood samples for serum trastuzumab concentration were collected at peak (post-infusion) and trough (preinfusion) time points in cycles 1–13 for the IV group and at trough time points for the SC group. Additional PK samples were collected at cycle 7 (during neoadjuvant treatment) and cycle 12 (during adjuvant treatment) for both groups to determine the concentration–time profile for noncompartmental PK analysis and comparison of PK during neoadjuvant and adjuvant treatment phases (Online Resource 2). All PK data from cycles 1 to 13 were used in this analysis. Results from the phase I study (NCT00800436) were not included in this analysis because that study included as patients with EBC and healthy male volunteers were present, and not all data from the relevant covariates were collected.

### Efficacy data included in the analysis

pCR was defined as “absence of invasive neoplastic cells in the breast.” Since the noninferiority hypothesis was to be tested, the per-protocol population was the primary analysis population.

### Safety data included in the analysis

AEs were graded according to National Cancer Institute Common Terminology Criteria for Adverse Events (NCI-CTCAE V3.0) [15].

### Serum trastuzumab concentration assays

Serum trastuzumab concentrations were determined using a validated receptor binding, enzyme-linked immunosorbent assay (ELISA). The lower limit of quantification (LLOQ) of the assay is 0.156 μg/mL [16].

### PK data handling

Patients were defined as evaluable for PK analysis if they had at least one adequately documented dose of trastuzumab and a corresponding PK sample. Records were excluded if the time of drug administration or sample collection was missing.

Observations below the LLOQ were omitted. A more sophisticated analysis method, e.g., a censored-data likelihood method, was not deemed necessary for HannaH, since it was a multiple dosing study where most PK samples were at steady state with concentrations >20 µg/mL (substantially higher than the LLOQ of 0.156 μg/mL).

Outliers were identified using an initial two-compartment model with linear elimination from the central compartment, first-order absorption of the SC regimen, and a proportional plus additive residual error model. Data with weighted residuals (WRES) >3 or conditional weighted residuals (CWRES) >3 of the initial model were considered to be potential outliers and omitted from the PopPK analysis. A sensitivity analysis, where the final model was reestimated using all data including outliers, was performed and the parameter estimates compared. Observations below the minimum quantifiable concentration limit were also omitted from the PopPK analysis.

### PopPK analysis

The PopPK methods were based on the FDA’s *Guidance for Industry: Population Pharmacokinetics* [17]. Trastuzumab PK data were analyzed by nonlinear mixed-effects modeling with NONMEM version 7.1.2 (ICON Development Solutions, Ellicott City, MD, USA) using the first-order conditional estimation method with interaction (FOCEI). Perl-speaks-NONMEM (PsN, version 3.2, http://psn.sourceforge.net/) was used to aid the model development; S-PLUS (TIBCO Software Inc., Palo Alto, CA, USA) and R statistical software [18] were used for data assembly, exploratory data analysis, model diagnosis, and model simulations.

Models were evaluated based on the likelihood objective function value (OFV) provided by NONMEM, graphical evaluation, and precision of the parameter estimates (relative standard error [RSE]).

A two-step approach was used for covariate identification [19]. In the first step, a univariate screening of the covariates was conducted. Only the covariates that showed a significant (*p* < 0.01) effect on the estimated PK parameters and could be meaningfully explained from both a clinical and scientific perspective, were carried through to the second step. The second step was a stepwise, backward elimination process, starting with the full model and removing each covariate one at a time. The least significant covariate was removed, and the process was repeated. The level of significance to retain a covariate was *p* < 0.001. In the final model, all retained covariates are considered to be statistically significant.

The effects of continuous covariates were modeled as follows:

$$ \theta_{i} = \theta_{\text{pop}} \cdot \left( {\frac{{{\text{Cov}}_{i} }}{{{\text{Cov}}_{\text{pop}} }}} \right)^{{k_{\text{cov}} }} $$

The effects of categorical covariates were modeled as follows:

$$ \theta_{i} = \theta_{\text{pop}} \cdot {\text{e}}^{{k_{\text{cov}} X_{i} }} $$

where *θ* is a PK model parameter, Cov is a continuous covariate, *X* is an indicator variable for a categorical covariate, *i* is the index for the subject, pop is an index denoting the typical value in the population, and *k*_cov_ is a coefficient describing the strength of the covariate effect on the PK parameter.

Patient factors evaluated as covariates were selected based on potential clinical relevance and previous experience with trastuzumab and other monoclonal antibodies. For CL, these were: patient demographics (age, baseline body weight, race), baseline laboratory measurements [alkaline phosphatase (ALK), serum albumin (ALBU), total bilirubin (TBIL), aspartate transaminase (AST), alanine transaminase (ALT), creatinine clearance (CrCL)], Eastern Cooperative Oncology Group (ECOG) performance status, treatment setting (neoadjuvant or adjuvant), HER2 overexpression level, and immunogenicity variables [anti-trastuzumab antibodies (ATAs) and anti-hyaluronidase antibodies (AHAs)]. For *V*_c_, these were: patient demographics, ECOG performance status, and HER2 overexpression level. For peripheral volume of distribution (*V*_p_), these were: baseline body weight, race, and HER2 overexpression level. Where covariate data were missing for ≤10 % of the patients, continuous covariates were imputed as the population median and categorical covariates were categorized as the reference category.

The final PK model was evaluated using internal validation procedures: nonparametric bootstrap resampling techniques (*n* = 1000 trial replicates) stratified by treatment arm and visual and numerical predictive checks [20] (*n* = 1000) stratified by treatment arm.

The extent of shrinkage derived from the final model was assessed for each between-subject variability term (*η*), as well as for residual error (*ε*). High *η* shrinkage indicates a lack of information for a reliable estimate of the individual empirical Bayes PK parameter estimates (EBEs); interpretation of EBEs should be approached with caution whenever substantial *η*- or *ε*-shrinkage exists (e.g., >30 %) [21].

### Evaluation of body weight impact on PK parameters

The impact of baseline body weight on the predicted steady-state trough concentration (*C*_min,ss_) and area under the curve (AUC_ss_) was evaluated with simulations from resampling of the EBEs. The simulation takes into account potential correlation of between-subject variability with covariates. In addition, stochastic simulations of 100 trials were performed using the observed set of covariate values of all patients from HannaH, to account for potential correlation among covariates, random variability between subjects, and random residual variability.

### PK exposure–response analysis

The relationship between pCR and *C*_min,ss_ was assessed using a multiple logistic regression (MLR) analysis for pCR with the covariates *C*_min,ss_, body weight, and treatment arm, and all interactions with treatment arm. Body weight was included in the analysis as the use of a fixed dose might result in higher exposure in lighter patients than in heavier patients; weight-based IV dosing was expected to result in higher exposure in heavier patients.

The relationship between grade ≥3 AEs and AUC_ss_ was assessed using MLR for incidence of grade ≥3 AEs and the covariates AUC_ss_, body weight, treatment arm, and all interactions with the treatment arm.

The estimated odds ratios and corresponding 95 % CIs from the fitted models were graphically represented for patients with specified body weights and model-predicted PK parameters. These values were determined by selecting the first quartile (25th percentile), second quartile (median), and third quartile (75th percentile) of the population distribution.

## Results

### Patient population and PK samples

HannaH enrolled 596 patients, 595 of whom received at least one dose of trastuzumab (297 patients in the SC arm and 298 patients in the IV arm). There were 6403 PK samples collected in the SC arm [including 95 (1.5 %) outliers from 72 patients] and 9790 samples collected in the IV arm [including 337 (3.4 %) outliers from 138 patients]. Typically, only one or two samples per patient (out of 24 and 36 planned samples per patient in the SC and IV arms, respectively) were excluded during the 13 treatment cycles. All data from three patients were excluded because >10 samples from each of these patients were outliers. All identified outliers were visually inspected, and it was confirmed that there was no bias against low versus high trough concentrations. The identified outliers were mainly trough concentrations that were greater than the peak concentration and/or vice versa. The dataset for PK model development contained 592 patients and 15,761 trastuzumab serum PK samples. A final sensitivity analysis, where all data, including outliers, were included, was performed and resulted in similar parameter estimates. Patient demographics and laboratory and disease covariates are summarized in Online Resource 3.

### Trastuzumab PK

The final PK model (Online Resource 4) was a two-compartment model with parallel linear and nonlinear (Michaelis–Menten) elimination from the central compartment. SC absorption was modeled as a first-order process. PK parameter estimates for trastuzumab (Table 1) were well defined (RSE < 39 %), as assessed by a stratified nonparametric bootstrap procedure. Of the 1000 datasets generated for bootstrapping, 941 (94.1 %) runs minimized successfully. Between-subject variability was modeled using a log-normal variance model, and the residual error variability was described by a combined proportional plus additive residual error model.

**Table 1:** Final population pharmacokinetic parameters for trastuzumab

Parameter	Population estimate (% RSE)	Between-subject variability (CV %)	Bootstrap estimate, median (2.5, 97.5 percentile)
Bioavailability of SC regimen (−)	0.771 (1.45)	13.0	0.773 (0.747, 0.797)
First-order SC absorption rate [Ka (day^−1^)]	0.404 (2.92)	–	0.403 (0.381, 0.429)
Linear CL (L/day)	0.111 (10.3)	30.0	0.115 (0.094, 0.153)
*V*_max_ (mg/day)	11.9 (19.9)	–	11.0 (5.2, 16.3)
Km (mg/L)	33.9 (38.6)	–	28.5 (5.69, 67.2)
*V*_c_ (L)	2.91 (1.24)	19.1	2.92 (2.85, 3.00)
*Q* (L/day)	0.445 (10.5)	–	0.444 (0.314, 0.539)
*V*_p_ (L)	3.06 (3.23)	50.4	3.08 (2.88, 3.31)
Influence of body weight on linear CL	1.04 (11.3)	–	0.998 (0.695, 1.26)
Influence of body weight on *V*_c_	0.443 (11.3)	–	0.445 (0.336, 0.535)
Influence of body weight on *V*_p_	0.500 (22.2)	–	0.481 (0.200, 0.735)
Influence of ALT on linear CL	0.144 (20.8)	–	0.136 (0.085, 0.205)
Proportional variability (%)	23.9 (3.62)	–	23.9 (23.0, 24.7)
Additive variability (µg/mL)	4.48 (21.7)	–	4.43 (3.33, 5.27)

ALT, alanine transaminase; CV, coefficient of variation; Km, concentration at which the nonlinear clearance rate is half of *V*_max_; RSE, relative standard error; SC, subcutaneous

CL_*i*_ = 0.111 · (WT_*i*_/68)^1.04^ · (ALT_*i*_/19)^0.144^

$$ V_{{{\text{c}}_{i} }} = 2.91 \cdot \left( {{\text{WT}}_{i} /68} \right)^{0.443} $$

$$ V_{{{\text{p}}_{{_{i} }} }} = 3.06 \cdot \left( {\text{WT}_{i} /68} \right)^{0.500} $$

Goodness-of-fit plots showed good agreement between predicted and observed concentrations, with no bias in residuals over time and across predicted concentration values (Online Resource 5). There was an acceptable level of shrinkage for all trastuzumab PK parameters: 11.5 % for CL, 22.3 % for *V*_c_, and 19.1 % for *V*_p_.

**Fig. 1:**
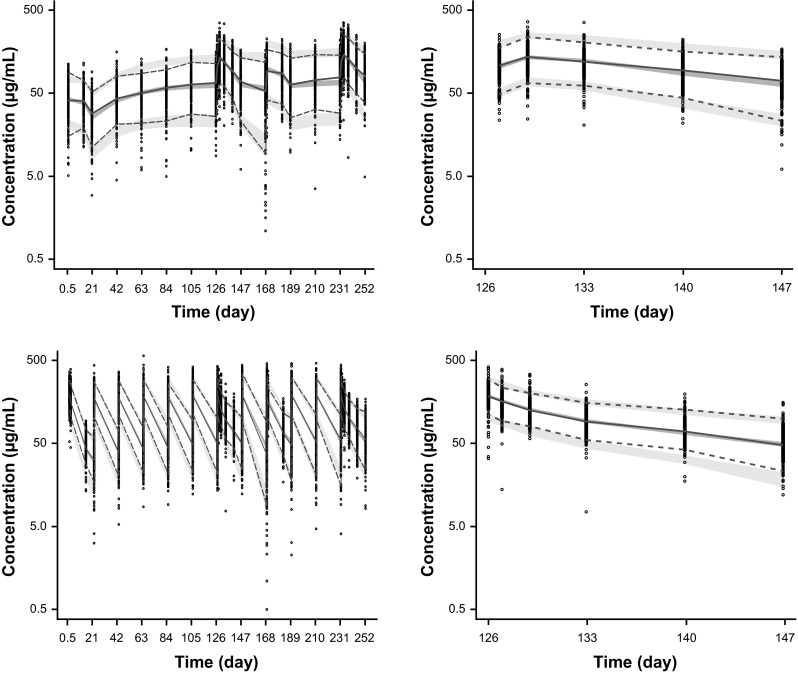
Simulated and observed concentrations with the SC (*top*) and IV (*bottom*) regimens. IV, intravenous; SC, subcutaneous. *Circles* are observed trastuzumab serum concentrations; *solid lines* represent the median observed values; *dashed lines* represent 5 and 95 % prediction intervals of the observed values; *shaded areas* represent the median predicted values or the spread (5 and 95 %) of the predicted concentrations; the width of the *shaded areas* indicate 95 % CIs; *left panel* shows the entire time course while the *right panel* shows cycle 7 only

Visual predictive checks stratified by administration (SC or IV) evaluated the ability of the model to reproduce the distribution of the observed PK data. The visual predictive check (Fig. 1) showed that the model mimics the central tendency and the spread of the PK in both the SC and IV populations. The plots were also stratified by quartiles of baseline body weight, and the model matched the PK of patients with low or high body weight. In the numerical predictive check calculation, the 5th, 25th, 50th, 75th, and 95th percentiles of the model predictions corresponded to the 4, 21, 50, 77, and 95 % quartiles of the observed concentrations, respectively. Therefore, both visual and numerical predictive checks suggested that the model adequately describes the distribution of trastuzumab PK concentrations.

At clinically relevant steady-state concentrations (Online Resource 6), total CL was estimated to decrease from trough to peak serum concentrations: 0.22–0.18 L/day CL for trough/peak concentrations of 75–148 µg/mL (SC regimen); 0.24–0.17 L/day CL for trough/peak concentrations of 57–170 µg/mL (IV regimen). Model simulations determined that 90 % of steady-state PK (*C*_min,ss_) was achieved by approximately cycle 6 (126 days) and cycle 5 (105 days) for the SC and IV regimens, respectively. *C*_min_ reached steady state later than *C*_max_ and AUC because the impact of nonlinear clearance is more significant at low concentrations.

### Impact of patient characteristics and pathophysiology on trastuzumab PK

CL patient characteristics included: patient demographics (age, baseline body weight, and race), baseline laboratory measurements (ALK, ALBU, TBIL, AST, ALT, and CrCL), ECOG performance status, treatment setting (neoadjuvant or adjuvant), HER2 overexpression level, and immunogenicity variables (ATAs and AHAs). For *V*_c_, these were: patient demographics, ECOG performance status, and HER2 overexpression level. For *V*_p_, these were: baseline body weight, race, and HER2 overexpression level.

The effect of patient demographics, baseline laboratory measurements, renal function (CrCL), immunogenicity, and disease factors on PK parameters was assessed. The covariates identified in the univariate screening step to have a significant (*p* < 0.01) impact on PK included: body weight, ALT, ALBU, CrCL, TBIL, and ALK on CL; body weight and race on *V*_c_; body weight on *V*_p_. Following the backward elimination step, the only two covariates remaining with a significant (*p* < 0.001) impact on PK were body weight (increased body weight resulted in increased CL and *V*_c_) and ALT (increased ALT resulted in increased CL); parameter–covariate relationships included in the final model are given in Table 1.

After inclusion of body weight and ALT in the final model, between-subject variability decreased from 31.3 to 30.0 % for linear CL, from 20.1 to 19.1 % for *V*_c_, and from 59.4 to 50.4 % for *V*_p_, compared with the base model without covariates. Therefore, 8 % of the total variability in linear CL was explained by body weight and ALT, 10 % of the variability in *V*_c_ was explained by body weight, and 28 % of the variability in *V*_p_ was explained by body weight. Inclusion of the covariates did not result in any change in the residual error magnitude.

A sensitivity analysis was performed to examine the influence of covariates on trastuzumab exposure (*C*_min,ss_ and AUC_ss_) with the SC regimen (Online Resource 7). Predicted *C*_min,ss_ varied from 35 to 123 μg/mL (5th–95th percentiles) across the overall patient population, representing a −54 to +63 % change from the reference patient [*C*_min,ss_ 75.4 μg/mL (with body weight 68 kg and ALT 19 IU/L)], while variability in body weight resulted in a −31 to +39 % change. The effect of ALT on *C*_min,ss_ was minimal, within the range of −14 to +13 %. The effect of body weight and ALT showed a similar trend for AUC_ss_.

No other tested covariates, including demographic variables (age and race), other laboratory variables reflecting hepatic function (ALBU, AST, and TBIL) or renal function (CrCL), ALK, ECOG performance status, HER2 expression level, antidrug antibodies (ADAs; including both ATAs and AHAs) or disease status (neoadjuvant vs. adjuvant) had statistically significant effects on trastuzumab PK.

### Impact of body weight on exposure with SC and IV regimens

**Fig. 2:**
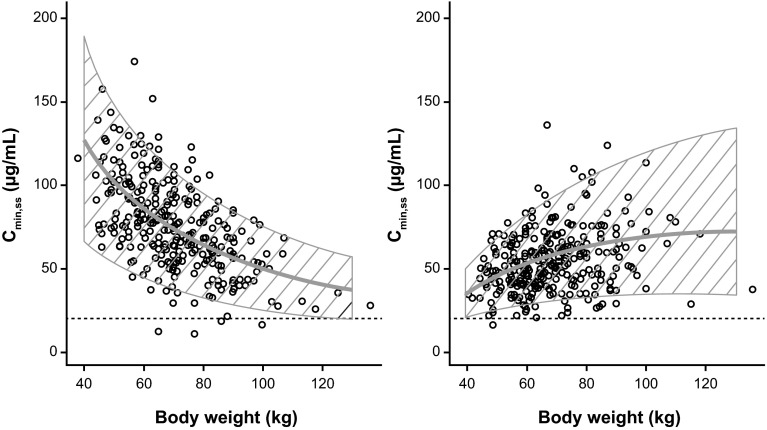
Impact of body weight on *C*_min,ss_ for the fixed-dose SC regimen (*left*) and the weight-based IV regimen (*right*). *C*_min,ss_, steady-state trough concentration; IV, intravenous; q3w, every 3 weeks; SC, subcutaneous. *C*_min,ss_ following administration of a fixed 600 mg dose SC q3w regimen (*left*) and a weight-based 8 mg/kg loading dose, 6 mg/kg maintenance dose IV q3w regimen (*right*). *Circles* are the individual model-predicted *C*_min,ss_ values for patients in the HannaH study. The *solid line* shows the impact of body weight on *C*_min,ss_ in patients with typical pharmacokinetics. The *dashed area* represents the spread (5 and 95 %) based on stochastic simulations (*n* = 300 for each body weight value), including between-subject variability. The *dotted horizontal line* is the 20 μg/mL target *C*_min,ss_ based on preclinical xenograft efficacy models

Since the IV regimen was weight-adjusted, patients with greater baseline body weight received a greater absolute dose. Weight-adjusted dosing overcompensates for the effect of body weight on trastuzumab PK and results in greater exposure in heavier patients. The effect is reversed in patients receiving the SC regimen (lesser exposure for heavier patients) because patients received a fixed dose. In spite of the impact of body weight on exposure, there was a large overlap in the distribution of *C*_min,ss_ and AUC_ss_ across the body weight range, for both regimens. Trastuzumab concentrations in heavier SC patients were similar or higher when compared with all body weight ranges or groups of IV patients (Fig. 2; Online Resource 8). Furthermore, most patients (98 % for SC and 99.5 % for IV) achieved or exceeded the historical target *C*_min,ss_ of 20 μg/mL (based on preclinical xenograft efficacy models) [12].

### Impact of regimen on PK

**Fig. 3:**
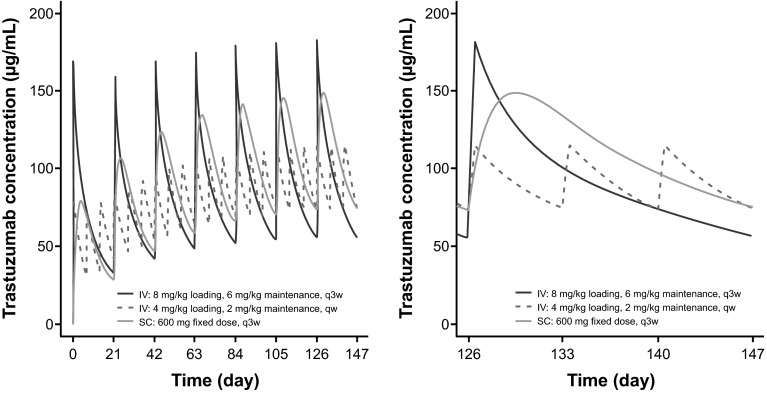
Model-predicted concentration–time profiles for the approved SC and IV regimens. ALT, alanine transaminase; IV, intravenous; q3w, every 3 weeks; SC, subcutaneous. Concentration–time profiles are simulated for a 600 mg fixed-dose q3w SC regimen, an 8/6 mg/kg q3w IV regimen and a 4/2 mg/kg qw IV regimen, using estimated population pharmacokinetic parameters for a typical patient (with body weight 68 kg and ALT 19 IU/L, as provided in Table 1. Inter-individual variability and model residual error were not included in the simulation). Simulation was performed for seven cycles. *Left panel* shows the profile from cycles 1 to 7; *right panel* shows cycle 7 only

Typical predicted trastuzumab concentration–time profiles were compared for three regimens: IV (8 mg/kg loading dose followed by 6 mg/kg q3w, 4 mg/kg loading dose followed by 2 mg/kg qw) and SC (fixed 600 mg q3w) (Fig. 3). The simulation comprised reference patients with body weight 68 kg and ALT 19 IU/L. At steady state (cycle 7, right panel), the SC regimen produced greater trastuzumab concentrations than the IV regimens during the majority of treatment cycles, while the q3w IV regimen (8/6 mg/kg) had a higher peak concentration. The peak concentration (*C*_max,ss_) of the SC regimen at steady state was reached around 4 days post-dose. Predicted *C*_min,ss_ was 57, 75, and 75 µg/mL for the IV 8/6 mg/kg q3w, IV 4/2 mg/kg qw, and SC 600 mg q3w dosing regimens, respectively (Online Resource 9). The higher *C*_min,ss_ for SC dosing is attributed to the 600 mg fixed dose selected and, for the qw regimen, to a lesser effect of the nonlinear clearance component than that seen in the q3w schedule. The predicted *C*_max,ss_ was 182, 116, and 149 µg/mL for the three dosing regimens, with the lower C_max_ of the SC 600 mg q3w regimen and the IV 4/2 mg/kg qw regimen reflecting the longer absorption period of the SC regimen and the lower dose of the IV 4/2 mg/kg qw regimen, respectively. The predicted AUC_ss_ over a 3-week period was similar between regimens, with values of 1994, 1951, and 2337 µg day/mL for the IV 8/6 mg/kg q3w, IV 4/2 mg/kg qw, and SC 600 mg q3w dosing regimens, respectively.

### PK exposure–response relationships

The per-protocol population included in the exposure–response analyses comprised 260 and 263 patients in the SC and IV arms, respectively. pCR rates were similar for the IV and SC arms irrespective of body weight or quartiles of predicted exposure, with the exception of the lowest body weight quartile (<58 kg) and the second highest *C*_min,ss_ quartile (≥61.5 μg/mL, <78.2 μg/mL), where pCR rates [54 % (*n* = 56) and 53 % (*n* = 73), respectively] were higher in the SC arm (Table 2; Online Resource 10); while this analysis is limited by the small sample sizes of the subgroups, pCR rates were similar across *C*_min,ss_ and AUC_ss_ quartiles, for both arms.

**Table 2:** Summary of efficacy (pCR) and safety (grade ≥3 AE) by body weight and exposure quartiles

pCR	IV trastuzumab *n* = 263		SC trastuzumab *n* = 260	
	Patients in subgroup (*n*)	Responders [*n* (%)]	Patients in subgroup (*n*)	Responders [*n* (%)]
Baseline body weight quartile (kg)				
<58	62	23 (37)	56	30 (54)
≥58, <67	74	32 (43)	63	28 (44)
≥67, <79	68	28 (41)	68	31 (46)
≥79	59	24 (41)	73	29 (40)
Predicted *C*_min,ss_ quartile (μg/mL)				
<45.9	97	41 (42)	33	12 (36)
≥45.9, <61.5	84	36 (43)	45	16 (36)
≥61.5, <78.2	57	20 (35)	73	39 (53)
≥78.2	22	9 (41)	109	51 (47)
Missing	3	1 (33)	0	0

Grade ≥3 AEs	IV trastuzumab *n* = 298		SC trastuzumab *n* = 297	
	Patients in subgroup (*n*)	Patients with AEs [*n* (%)]	Patients in subgroup (*n*)	Patients with AEs [*n* (%)]
Baseline body weight quartile (kg)				
<59	77	50 (65)	71	37 (52)
≥59, <68	84	42 (50)	70	37 (53)
≥68, <79	70	31 (44)	71	42 (59)
≥79	67	33 (49)	85	43 (51)
Predicted AUC_ss_ quartile (μg day/mL)				
<1710	101	55 (54)	47	27 (57)
≥1710, <2055	94	51 (54)	52	25 (48)
≥2055, <2479	69	31 (45)	81	45 (56)
≥2479	31	17 (55)	117	62 (53)
Missing	3	2 (68)	0	0

Exposure subgroups are according to predicted values from the final population pharmacokinetic model

AUC_ss_, steady-state area under the curve; *C*_min,ss_, steady-state trough concentration

**Fig. 4:**
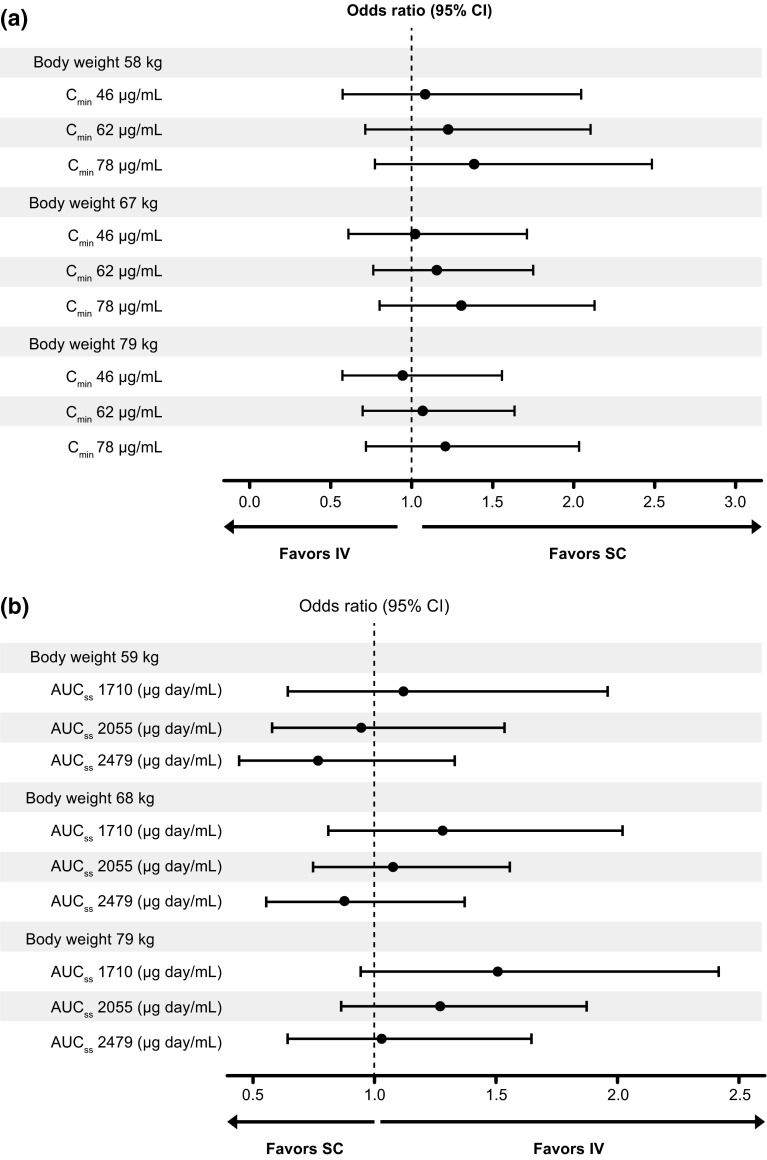
Odds ratios for **a** pCR subgroups of body weight and predicted *C*_min,ss_ quartiles (per-protocol population) and **b** rates of grade ≥3 AEs of subgroups by body weight and predicted AUC_ss_ quartiles (safety population). AE, adverse event; AUC_ss_, steady-state area under the curve; *C*_min,ss_, steady-state trough concentration; IV, intravenous; pCR, pathologic complete response; SC, subcutaneous

MLR showed that none of the parameters investigated (*C*_min,ss_, body weight, treatment arm, and interaction terms with treatment arm) impacted the primary efficacy endpoint, pCR (*p* > 0.1 for all, Online Resource 11). The estimated odds ratios and corresponding 95 % CIs from the fitted model are graphically represented in Fig. 4a for patients with specified body weights (59, 68, and 80 kg) and model-predicted *C*_min,ss_ values (46, 62, and 78 μg/mL). The estimated odds ratios for pCR were similar in each subgroup, with overlapping CIs. Hence, no statistically relevant impact of predicted *C*_min,ss_ or body weight on pCR was observed.

The safety population in the exposure–response analyses comprised 297 and 298 patients in the SC and IV arms, respectively. No clinically meaningful pattern was observed in grade ≥3 AE rates within the quartile groups of model-predicted *C*_min,ss_, AUC_ss_, or body weight quartile groups in either the SC or IV treatment groups (Table 2; Online Resource 12). Rates were generally comparable between the two arms, and there was no pattern suggestive of an increase in grade ≥3 AEs with increasing exposure or decreasing body weight [14].

MLRs for grade ≥3 AEs demonstrated that none of the covariates had a significant effect on the probability of grade ≥3 AEs (*p* values of parameter estimates were >0.05, Online Resource 13). The estimated odds ratios for grade ≥3 AEs were similar in each subgroup, with overlapping CIs (Fig. 4b). Hence, no statistically relevant impact of predicted AUC_ss_ or body weight on occurrence of grade ≥3 AE was observed.

## Discussion

Trastuzumab PK was evaluated in 595 female patients with HER2-positive EBC receiving trastuzumab in the neoadjuvant and adjuvant settings in the phase III HannaH study using a population modeling approach. In addition, the relationship between trastuzumab exposure, efficacy (pCR), and safety (grade ≥3 AEs) during neoadjuvant treatment was evaluated. Patients were randomized to receive either a fixed 600 mg SC regimen (q3w) or the IV regimen (8 mg/kg loading dose followed by 6 mg/kg q3w). Overall, while PopPK analysis showed body weight and ALT had an influence on PK, their effects were considered to be of a nonclinically relevant magnitude. This was confirmed by the PK exposure–efficacy–safety analysis showing lack of a distinct relationship between *C*_min,ss_, trastuzumab regimen and route of administration, or body weight and pCR or AUC_ss_, trastuzumab regimen and route of administration, or body weight with grade ≥3 AEs.

In this PopPK analysis, SC and IV trastuzumab serum concentration–time data were pooled together and described by a two-compartment model, with first-order absorption following SC injection and two parallel linear and nonlinear eliminations from the central compartment. The nonlinear CL, probably a result of target-mediated drug disposition, is consistent with previous reports for other monoclonal antibodies [11]. As a result of the nonlinear elimination pathway, trastuzumab elimination is faster and the half-life shorter at low compared with high concentrations, which is consistent with target-mediated CL that gets saturated at higher concentrations. There was a good agreement between predicted and observed concentrations, and the model mimicked the central tendency and spread of the PK of both SC and IV trastuzumab, adequately describing the distribution of concentrations. At typical steady-state concentrations, total CL fluctuates due to nonlinearity, as trough/peak concentrations fluctuate from 75 to 140 μg/mL (SC regimen) and 57 to 170 (IV regimen); total CL is approximately linear, with the range of 0.22–0.18 and 0.24–0.17 for the SC and IV regimens, respectively. These values are comparable to those seen previously, where trastuzumab PK of weekly IV administration was described using a two-compartment model with linear clearance (0.225 L/day) [8]. Furthermore, *V*_c_ (2.91 L) was consistent with the previous analysis (2.95 L) [8]. Both total CL and *V*_c_ were also consistent with typical values for other humanized IgG1 monoclonal antibodies [5, 22, 23].

Bioavailability of the SC regimen was estimated to be 77 % (1.45 % RSE), with between-subject variability of 13.0 and 52.2 % shrinkage. Within-patient PK comparison of SC and IV data was not obtained in this study; however, the estimated bioavailability was comparable to that seen with data from the phase I study (NCT00800436), which included four patients who received both SC and IV trastuzumab; SC bioavailability in this study was estimated to be 0.873 (6.7 % RSE), with between-subject variability of 16.9 %, using a population modeling approach [10].

The impact of demographic and pathophysiological covariates on the PK of trastuzumab was investigated. Baseline body weight and ALT were identified as covariates that had statistically significant effects on trastuzumab PK (*p* < 0.001). Body weight was shown to influence the linear CL, *V*_c_, and *V*_p_ of trastuzumab, all of which increased with increasing body weight. This body weight effect is consistent with the previously reported covariate relationships for trastuzumab in patients with MBC [8]. Of note, higher ALT (typically linked to slower drug clearance) was associated with a higher clearance. The reasons for this finding are unknown, and it has also been observed with other monoclonal antibodies [6].

Sensitivity analyses assessed the impact of body weight and ALT on trastuzumab exposure and showed that while body weight had a modest effect on trastuzumab exposure, ALT had a minimal effect. In addition, body weight and ALT combined explained <10 % of between-subject variability in linear CL. Although body weight had an impact on trastuzumab exposure in the sensitivity analysis, the clinical trial simulations confirm previous findings that heavier patients who receive the fixed SC dose do not have compromised PK exposure compared with patients treated with the weight-adjusted IV regimen [10]. While the IV regimen overcompensates for body weight, resulting in higher *C*_min,ss_ in heavier patients, the reverse is observed for the fixed SC regimen (lower *C*_min,ss_ in heavier patients). However, the overall distribution of *C*_min,ss_ and AUC_ss_ in the entire population was similar for both dosing regimens. Additionally, the SC regimen achieved similar *C*_min,ss_ compared with the IV regimen in overweight patients, and most patients (98 %, 230/234) achieved the target trough concentration of 20 μg/mL (identified as having anti-tumor activity in preclinical efficacy models [12]) at steady state. Thus, neither body weight nor ALT is considered to be clinically relevant in the EBC population, as they would not necessitate dose adjustment.

The initial covariate screening based on the PopPK model showed that ALBU had a significant effect on CL (*p* = 0.0003). Following the backward elimination step, the effect of ALBU was not significant (*p* > 0.001) although the range was narrow (33–60 g/L) (Online Resource 6). ALBU level has been inversely associated with the clearance of other IgG antibodies [24]; however, in the current analysis, despite showing a trend toward an effect, it did not meet the predefined statistical criteria for selection as a significant covariate. It is hypothesized that ALBU level correlates with disease status, whereby patients with more severe disease have lower ALBU levels and clear IgG more quickly. The lack of an ALBU effect in HannaH may be attributed to the observed narrow range of relatively normal ALBU levels (33–60 g/L) in the EBC population studied, compared with the wider range of those in a more diverse population (such as patients with MBC). Renal function (CrCL) had a marginal effect on CL (*p* < 0.01 in the univariate covariate screening), but was not a significant covariate in the final model (*p* > 0.001), which may be a consequence of the body weight component used in the calculation of CrCL, as monoclonal antibodies typically do not have a renal clearance component [25, 26]. The development of ADAs (including both ATAs and AHAs) did not exhibit statistically significant effects on trastuzumab PK. However, due to the low numbers of ADA-positive patients (5.0 % ATA-positive and 5.7 % AHA-positive), these results should be interpreted with caution.

## Conclusion

For patients with HER2-positive EBC, a fixed dose of 600 mg SC trastuzumab q3w provides the desired PK exposure, with steady-state trough concentrations consistently above the historical efficacy target (20 μg/mL), regardless of race, body weight, and treatment setting (neoadjuvant or adjuvant). Within the ranges studied, the assessed patient covariates, including body weight, do not influence trastuzumab PK to a magnitude that would require a dose adjustment. The SC dose is further supported by the exposure–response analyses of pCR and grade ≥3 AEs, which did not identify a statistically meaningful impact of either body weight, exposure, or route of administration. Similar trastuzumab exposure is expected in patients with MBC and supports the appropriateness of a fixed 600 mg SC dose across breast cancer indications.

## Electronic supplementary material

Supplementary material 1 (DOCX 771 kb)
