# Supplementary material for: Population pharmacokinetic and exposure–response analysis for trastuzumab administered using a subcutaneous “manual syringe” injection or intravenously in women with HER2-positive early breast cancer
Source: Cancer Chemother Pharmacol. 2015 Dec 8;77:77–88. doi: 10.1007/s00280-015-2922-5 (PMC4706584; doi:10.1007/s00280-015-2922-5)
Supplement: Supplementary file 1 — Supplementary material 1 (DOCX 771 kb) [file 280_2015_2922_MOESM1_ESM.docx]

Online Resource 1. Study design of the HannaH trial.


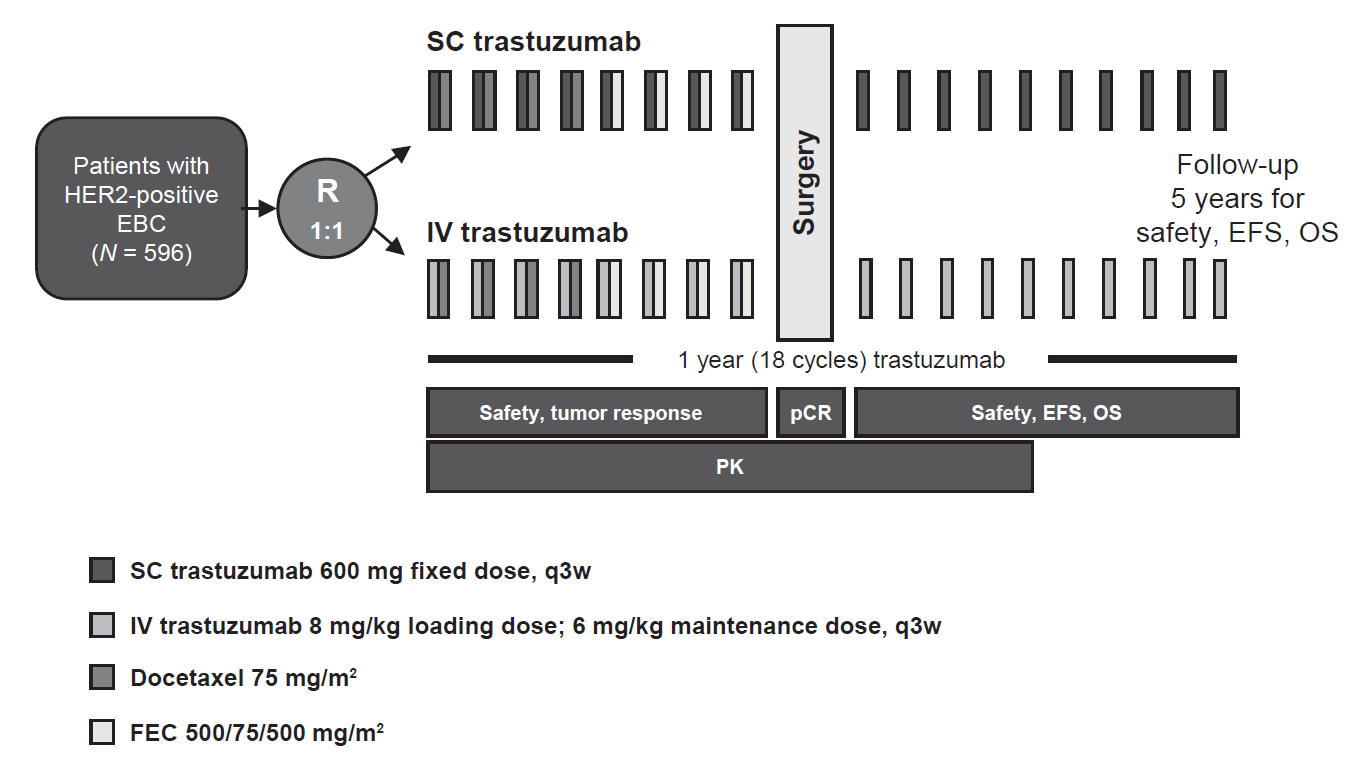


EBC, early breast cancer; EFS, event-free survival; FEC, fluorouracil/epirubicin/cyclophosphamide; IV, intravenous; OS, overall survival; pCR, pathologic complete response; PK, pharmacokinetics; SC, subcutaneous

Online Resource 2. Dose regimens and PK sampling schedules.

| **Route** | **Dose** | **Regimen** | **PK sample times** | **Patients, *n*** |
| --- | --- | --- | --- | --- |
| SC | 600 mg | q3w | Cycles 1 and 9: pre-dose, days 1 and 15 post-dose;  Cycles 2–6, 8, 10, 11, and 13: pre-dose;  Cycles 7 and 12: pre-dose, days 1, 2, 4, 8, and 15 post-dose. | 297 |
| IV | 8 mg/kg loading + 6 mg/kg | q3w | Cycles 1 and 9: pre-dose, end of IV infusion, days 2 and 15 post-dose;  Cycles 2–6, 8, 10, 11, and 13: pre-dose and end of IV infusion;  Cycles 7 and 12: pre-dose, end of IV infusion and days 2, 4, 8, and 15 post-dose. | 298 |

IV, intravenous; SC, subcutaneous

Online Resource 3. Overview of demographics, laboratory, and disease covariates (PK population).

| **Administration** | **SC** | **IV** | **Total** |
| --- | --- | --- | --- |
| # Patients | 297 | 298 | 595 |
| # PK planned samples per patients  (pre-/post-surgery) | 24  (13/11) | 36  (21/15) | - |
| # PK samples  (pre-/post-surgery)* | 6403  (3656/2747) | 9790  (5953/3837) | 16193  (9609/6584) |
| Sex (F) | 297 | 298 | 595 |
| Race (W/B/A/I/O) | 200/10/64/3/20 | 208/6/61/3/20 | 408/16/125/6/40 |
| Age (years) | 51 (26 to 82) | 50 (24 to 78) | 51 (24 to 82) |
| Baseline body weight (kg) | 69 (38 to 136) | 66 (41 to 135.5) | 67.5 (38 to 136) |
| CrCL (mL/min) | 95.7 (45.7 to 261.4) | 91 (33 to 221.6) | 92.8 (33 to 261.4) |
| ALBU (g/L) | 43 (34 to 54) | 43 (33 to 60) | 43 (33 to 60) |
| ALK (IU/L) | 75 (22 to 340) | 72.5 (14 to 464) | 74 (14 to 464) |
| TBIL (mg/dL) | 0.54 (0.14 to 1.9) | 0.53 (0.16 to 1.66) | 0.53 (0.14 to 1.9) |
| AST (IU/L) | 21 (4.6 to 110.8) | 20 (6.4 to 58.6) | 20.2 (4.6 to 110.8) |
| ALT (IU/L) | 20 (2 to 215) | 19 (3.5 to 133) | 19 (2 to 215) |
| HER2 overexpression level (2+/3+/NA) | 44/250/3 | 52/245/1 | 96/495/4 |
| ECOG (0/1/NA) | 253/40/4 | 260/35/3 | 513/75/7 |
| ATA (N/Y/NA) | 275/20/2 | 285/10/3 | 560/30/5 |
| AHA (N/Y/NA) | 261/34/2 | 0/0/298 | 261/34/300 |

AHA, anti-hyaluronidase; ALBU, serum albumin; ALK, alkaline phosphatase; ALT, alanine transaminase; AST, aspartate transaminase; ATA, anti-trastuzumab antibodies; CrCL, creatinine clearance; ECOG, Eastern Cooperative Oncology Group; NA, not available due to missing data; PK, pharmacokinetic; TBIL, total bilirubin

Race categories: W, White; B, Black; A, Asian; I, American Indian or Alaska native; O, other
Number of patients with missing continuous covariates: *n* = 1 for CrCL, *n* = 15 for ALBU, *n* = 3 for TBIL, *n* = 4 for AST, *n* = 1 for ALT
* Does not include samples that were below the limit of quantification

Online Resource 4. Trastuzumab two-compartment PK model with parallel linear and nonlinear elimination in early breast cancer patients.


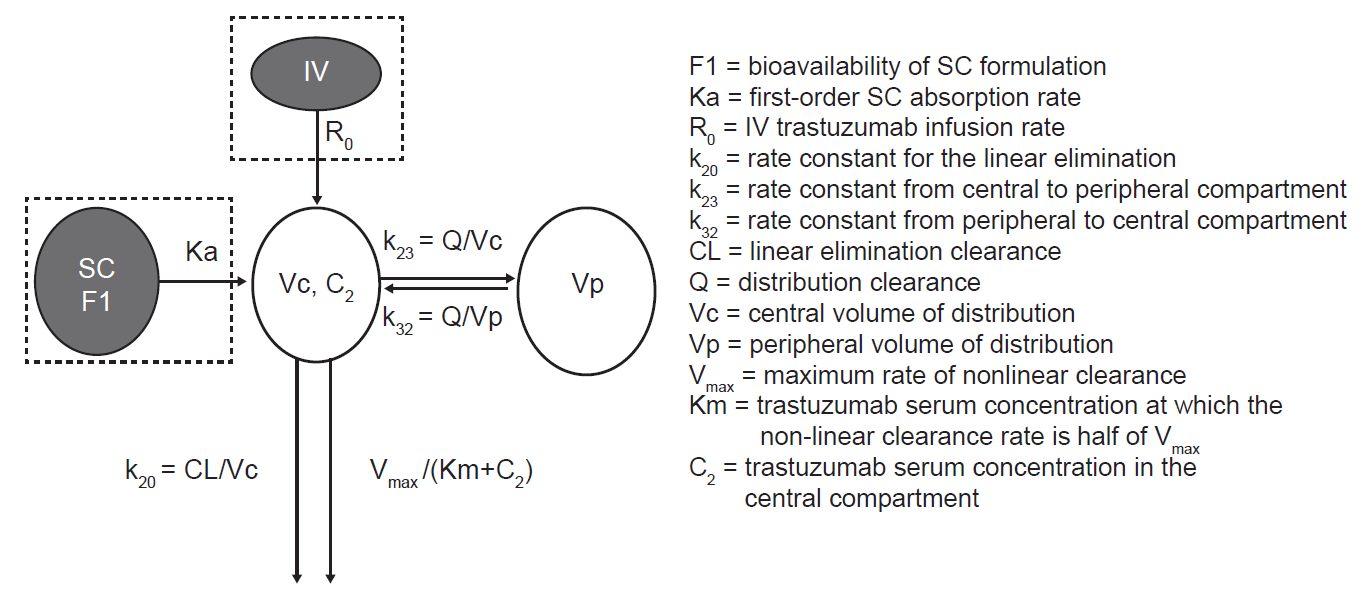


IV, intravenous; SC, subcutaneous

Online Resource 5. Final model goodness-of-fit plots.


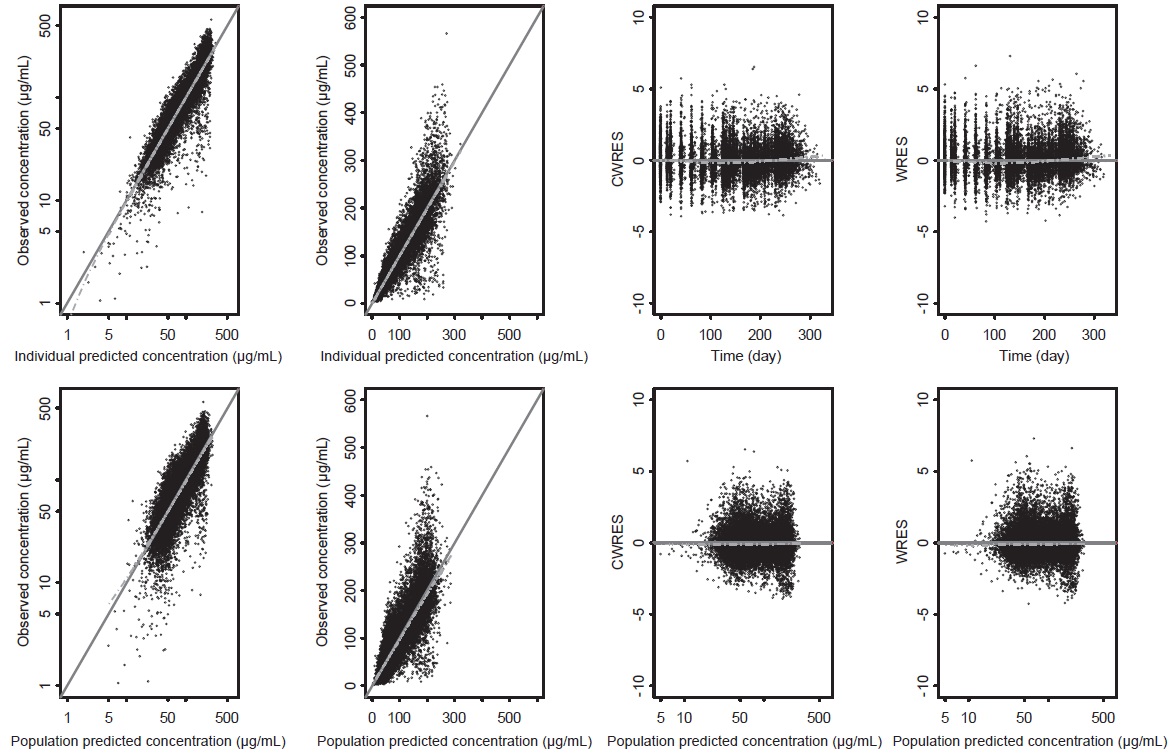


CWRES, conditional weighted residuals; PK, pharmacokinetic; WRES, weighted residuals

Top panel: Observed versus individual predicted concentrations (log-scale [left] and normal scale [middle left]), CWRES versus time (middle right), and WRES versus time (right) for the final PK model. Bottom panel: Observed versus population predicted concentrations (log-scale [left] and normal scale [middle left]), CWRES versus population predicted concentrations (middle right), and WRES versus population predicted concentrations (right) for the final PK model. Dashed lines are smooth (local smooth of scatter plot data) curves of the data.

Online Resource 6. Total clearance versus trastuzumab serum concentration.


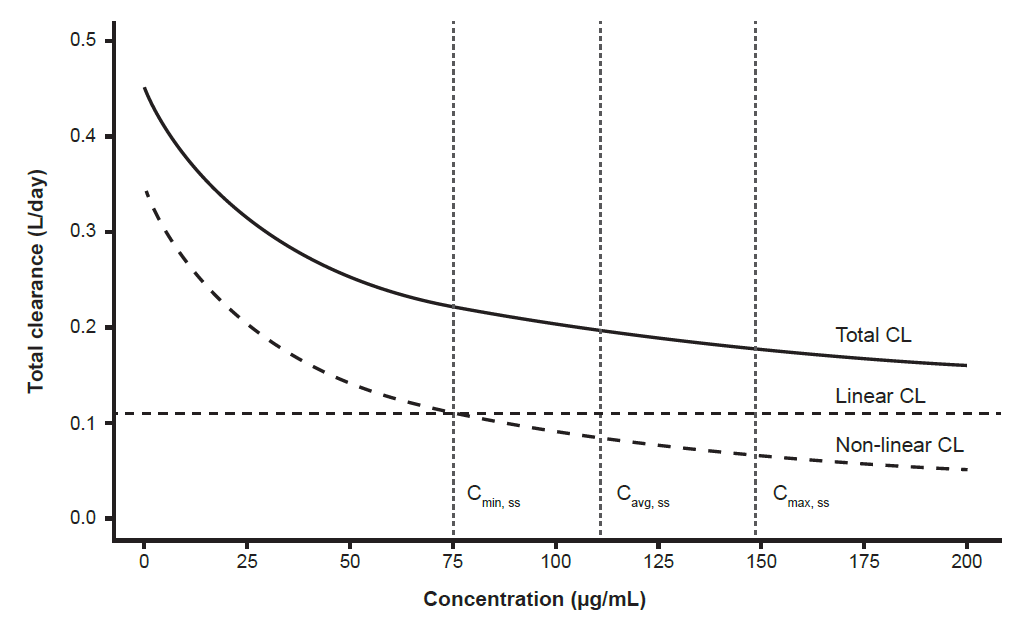


Vertical lines indicate steady-state C_max_, C_min_, and C_avg_ for the 600 mg q3w SC regimen.

Online Resource 7. Sensitivity plot comparing the effect of covariates on model-predicted exposure measures (C_min,ss_ and AUC_ss_) for the 600 mg q3w SC regimen*.*


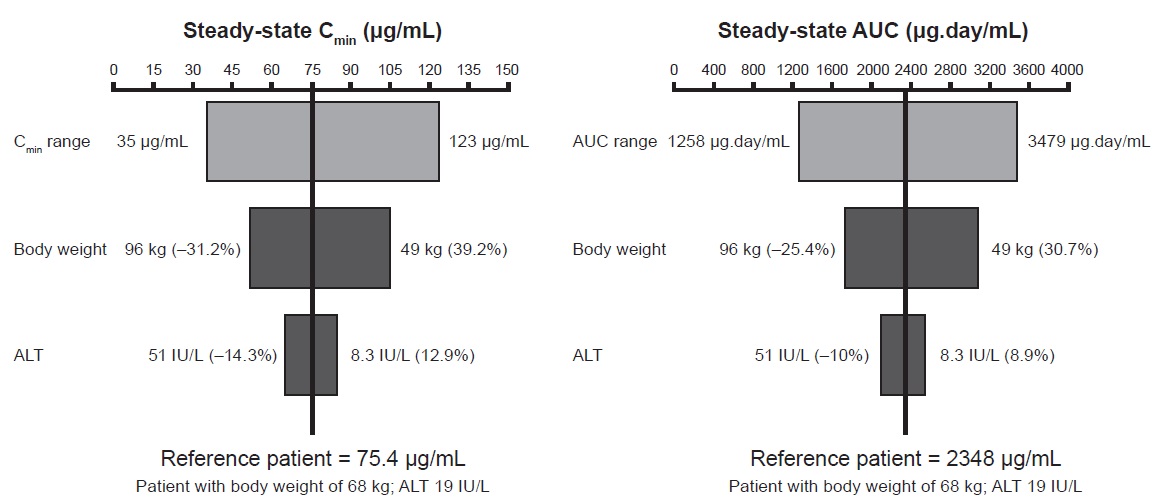


Each vertical reference line is the reference patient steady-state exposure value for the 600 mg q3w SC regimen. The top bar of each plot shows the 5^th^ to 95^th^ percentile of exposure values across the entire population, with the 5^th^ and 95^th^ percentile of each covariate and the percentage change from the typical exposure value labeled. The length of each bar describes the impact of that particular covariate on exposure.

Online Resource 8. Model-predicted exposure at steady state by baseline body weight after administration of the SC or IV q3w regimen.


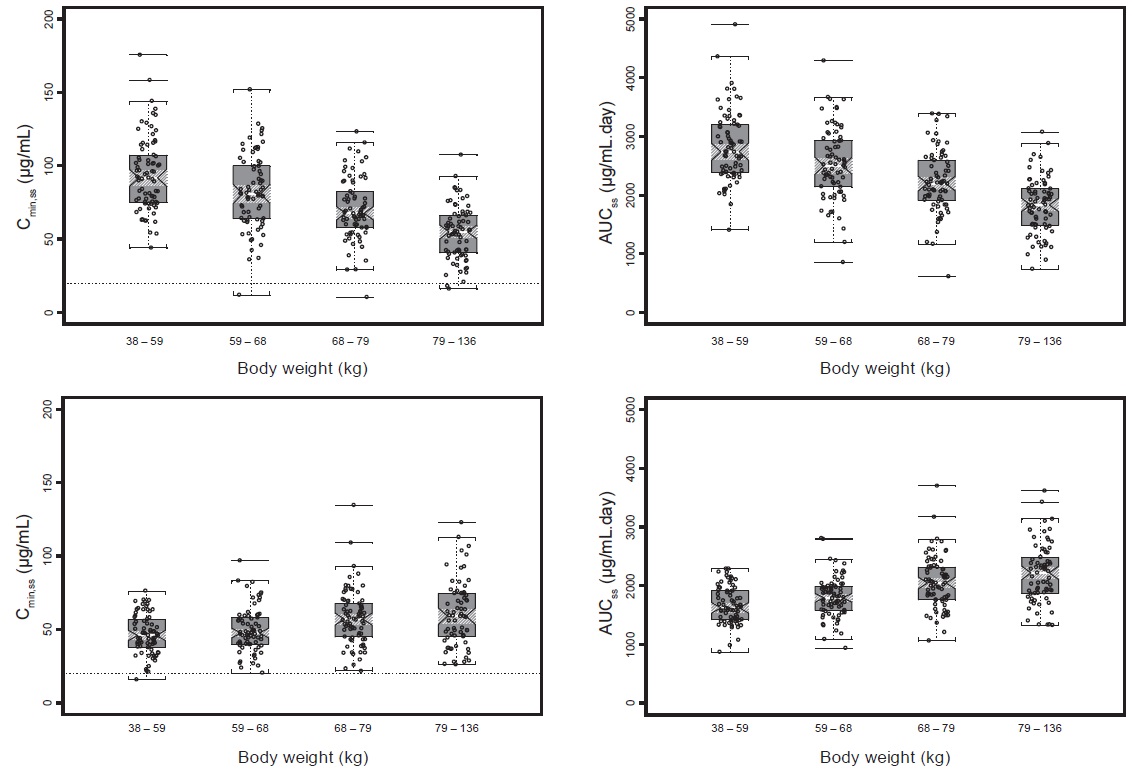


The PK variables were calculated using empirical Bayes (“post hoc”) PK parameters of the final nonlinear model for 600 mg SC q3w (top panels) and 8 mg/kg IV followed by 6 mg/kg IV q3w (bottom panels). The PK parameters were grouped by body weight distribution quartiles. The box shows the median, 25^th^ and 75^th^ percentiles of the patients. Shaded area represents the middle quartile and median. Lines outside of the upper and lower whisker indicate outliers. The dotted horizontal line is the 20 μg/mL target C_min,ss_ based on preclinical xenograft efficacy models.

Online Resource 9. Model-predicted steady-state exposures for the SC and IV regimens.

| Regimen | C_min,ss_ (μg/mL) | C_max,ss_ (μg/mL) | AUC_ss_  (μg•day/mL, per 3 weeks) |
| --- | --- | --- | --- |
| SC 600 mg fixed q3w | 75.0 (35.1–123.4) | 148.8 (86.1–213.6) | 2337.3 (1257.7–3478.1) |
| IV 8 mg/kg loading,  6 mg/kg maintenance q3w | 56.7 (27.4–83.2) | 182.3 (132.0–240.3) | 1994.0 (1324.4–2764.1) |
| IV 4 mg/kg loading,  2 mg/kg maintenance qw | 75.3 (44.6–107.6) | 115.7 (80.8–155.8) | 1950.9 (1298.1–2701.9) |

ALT, alanine transaminase; AUC_ss_, steady-state area under the curve; C_max,ss_, steady-state peak concentration; C_min,ss_, steady-state trough concentration; IV, intravenous; SC, subcutaneous
PK exposures are presented as typical, with the 5^th^ to 95^th^ percentile range obtained at Cycle 7. Typical PK exposure values were simulated using PK parameters for a typical patient (with body weight 68 kg and ALT 19 IU/L), as provided in Table 1, and the population ranges were simulated using the post hoc PK parameters of the population. Model residual error was not included in the simulation.

Online resource 10. Summary of pCR by exposure quartiles (per-protocol population).

|  | IV trastuzumab *n* = 263 | | SC trastuzumab  *n* = 260 | |
| --- | --- | --- | --- | --- |
|  | Patients in subgroup, *n* | Responders,  *n* (%) | Patients in subgroup, *n* | Responders,  *n* (%) |
| Predicted AUC_ss_ quartile (μg•day/mL)  <1720 ≥1720, <2055 ≥2055, <2462 ≥2462 Missing | 87 85 60 28 3 | 34 (39) 36 (42) 24 (40) 12 (43) 1 | 43 43 72 102 0 | 13 (30) 19 (44) 35 (49) 51 (50) 0 |

AUC_ss_, steady-state area under the curve

Online Resource 11. Multiple logistic regression for pathologic complete response and predicted steady-state trastuzumab exposure (C_min,ss_).

| Parameter | Estimate | Standard error | *P*-value |
| --- | --- | --- | --- |
| Intercept | –0.3543 | 0.5951 | 0.5517 |
| Arm (SC) | 0.0524 | 0.5951 | 0.9298 |
| Body weight (kg) | –0.00126 | 0.00660 | 0.8490 |
| C_min,ss_ (μg/mL) | 0.00190 | 0.00450 | 0.6728 |
| Body weight (kg)*arm (SC) | –0.00327 | 0.00660 | 0.6202 |
| C_min,ss_ (μg/mL)*arm (SC) | 0.00385 | 0.00450 | 0.3925 |

* Interaction between covariates
Estimate is the coefficient of logistic regression such that odds ratio = exponential of the estimate; *P*-value is computed based on Wald Chi-Square test. The *P*-value is computed based on Wald Chi-Square test.

Online Resource 12**.** Summary of grade ≥3 adverse events by exposure quartiles (safety population)

|  | IV trastuzumab *n* = 298 | | SC trastuzumab  *n* = 297 | |
| --- | --- | --- | --- | --- |
|  | Patients in subgroup, *n* | Patients with AEs, *n* (%) | Patients in subgroup, *n* | Patients with AEs, *n* (%) |
| Predicted C_min,ss_ quartile (μg/mL)  <45.8  ≥45.8, <61.1  ≥61.1, <78.4  ≥78.4  Missing | 109  98  64  24  3 | 60 (55)  48 (49)  33 (52)  13 (54)  2 | 37  52  84  124  0 | 21 (57)  28 (54)  42 (50)  68 (55)  0 |

C_min,ss_, steady-state trough concentration

Online Resource 13. Multiple logistic regression for incidence of grade ≥3 adverse events and predicted steady-state trastuzumab exposure (AUC_ss_).

| Parameter | Estimate | Standard error | *P*-value |
| --- | --- | --- | --- |
| Intercept | 0.8476 | 0.6053 | 0.1614 |
| ARM_SC | 0.0456 | 0.6053 | 0.9399 |
| WGTKG | –0.0113 | 0.00642 | 0.0777 |
| AUC | 0.000047 | 0.000179 | 0.7919 |
| WGTKG*ARM_SC | 0.00741 | 0.00642 | 0.2484 |
| AUC*ARM_SC | –0.00025 | 0.000179 | 0.1638 |

* Interaction between covariates
Estimate is the coefficient of logistic regression such that odds ratio = exponential of the estimate; *P*-value is computed based on Wald Chi-Square test.
